# Vibration suppression of scaled-down towers in offshore wind power based on pendulum tuned particle damper

**DOI:** 10.1371/journal.pone.0338380

**Published:** 2025-12-31

**Authors:** Wangqiang Xiao, Zhipeng Xie, Wuping Yao, Fei Mo, Zhiqin Cai

**Affiliations:** 1 School of Aerospace Engineering, Xiamen University, Xiamen, Fujian, China; 2 Shenzhen Research Institute of Xiamen University, Shenzhen, Guangdong, China; 3 Wuhan Second Ship Design and Research Institute, Wuhan, Hubei, China; 4 Amoy Institute of Technovation, Xiamen, Fujian, China; 5 Xiamen Quietime Co., Ltd., Xiamen, Fujian, China; Universiti Teknologi Malaysia, MALAYSIA

## Abstract

This paper presents a method for suppressing vibrations in a scaled wind turbine tower subjected to wind loads. The proposed approach employs a composite damping system—the Pendulum Tuned Particle Damper (PTPD). A mathematical model of the damping system was developed by deriving the equations of motion for the pendulum damper using Lagrange’s method, while accounting for the damping force exerted by particles. The optimal damper parameters were identified through numerical simulations. Through the method of the finite element-discrete element (FEM-DEM) coupled vibration reduction simulations tailored to the tower’s vibration characteristics, we demonstrated that 10 mm ferrous particles were optimal, resulting in an 81.1% vibration reduction. Experimental results confirmed the influence of the damper mass ratio, configuration, particle material, and particle size on damping effectiveness, thus validating the simulation methodology.

## 1. Introduction

Offshore wind power constitutes a significant renewable energy industry in China. Its development is crucial for optimizing the energy structure of coastal regions and achieving the national “carbon peak and carbon neutrality” objectives [1]. Driven by these policy goals, the offshore wind power sector is experiencing rapid growth. During the “14th Five-Year Plan” period, China’s newly installed offshore wind capacity is projected to reach nearly 40 million kW, establishing the country as the world’s largest offshore wind market [2].

Offshore wind turbines face harsher conditions than onshore systems, being subjected to complex forces such as wind, waves, ice, and currents [3]. During wind turbine operation, the tower-top vibration induced by wind loads exceeds that caused by wave loads. This difference is particularly pronounced under low wave conditions, where wave-induced effects become negligible compared to wind effects [4]. The development of larger offshore wind turbines has resulted in taller towers and longer blades. Vortex shedding from airflow generates periodic loading on both tower and blade surfaces. When the vortex shedding frequency approaches the structural natural frequency, vortex-induced resonance occurs, leading to significant vibrations and fatigue damage in the tower [5]. Although ice loading represents a major design consideration for turbines in cold regions, few wind farms in China experience sea ice effects. Notably, ice loading can excite the tower’s first-order mode similarly to wind loading [6]. The tower, as the primary load-bearing structure, ensures stable rotor operation at the design height. As the size of turbines continues to increase, so do the loads on these towers, placing higher demands on their stability, safety, and cost-effectiveness [7]. Currently, most offshore wind installations use monopile foundations in shallow waters (typically less than 60m deep), which account for more than 70% of global fixed-bottom installations [8,9]. However, monopile foundations have low structural damping, which impedes quick vibration attenuation and increases susceptibility to structural damage [10–12]. To address this, various damping systems have been developed worldwide to mitigate tower vibrations and extend their service life.

Liu et al. [13] applied a pendulum-type TMD to wind turbine towers to mitigate wind-induced vibrations, thereby reducing generator failures and structural damage. The influence of mass ratio, damping ratio, and natural frequency ratio of the pendulum-type TMD on vibration suppression and energy dissipation was systematically analyzed. To address tower fatigue induced by traditional Pendulum Tuned Mass Dampers (PTMDs) due to their large mass, Liu et al. [14] proposed a Pendulum Tuned Mass Damper Inerter (PTMDI) with corresponding design methodology. The PTMDI achieves superior control performance with equivalent added mass, enabling damper miniaturization. Yang et al. [15] investigated the use of inerter-based dynamic vibration absorbers (IDVAs) to mitigate wind-induced vibrations in a high-rise flue gas desulfurization tower. The performance of a conventional TMD and six IDVA configurations was compared in controlling tower vibrations. It was found that the two optimal IDVAs reduced the auxiliary mass by more than 34% while achieving a comparable vibration mitigation rate. This result demonstrates enhanced energy dissipation and a significant lightweight effect compared to the conventional TMD. Wang et al. [16] proposed a magnetically induced pendulum-tuned mass damper (PTMD) with tunable positive/negative stiffness to mitigate frequency detuning in conventional TMDs. Theoretical and experimental analyses indicate that the nonlinear magnetic forces introduce amplitude-dependent frequency characteristics. Although a slight reduction in control performance was observed, the retuned PTMD effectively broadens the frequency bandwidth and enables adaptive stroke control. Le et al. [17] analyzed the dynamic response of a monopile-supported turbine under combined wind and wave loads. Their designed Multiple Tuned Mass Dampers (MTMDs) demonstrated higher robustness than single TMDs, reducing tower-top acceleration and displacement standard deviations by over 50% while effectively lowering spectral response at the fundamental frequency. In another study, Le et al. [18] developed a Tuned Liquid Damper (TLD) for a 1:15 scale model of a 6.45 MW offshore wind turbine. Shake table tests showed the TLD reduced average acceleration response by 50% under sinusoidal excitation and achieved over 40% reduction in standard deviation under seismic excitation. Hemmati et al. [19] investigated hybrid vibration suppression using both TMD and Tuned Liquid Column Damper (TLCD) for a 5MW NREL offshore turbine under stochastic wind loads. Results showed higher TMD efficiency during operation, while the TLCD performed better during shut-down, validating the hybrid system’s effectiveness. Dong et al. [20] proposed a C-shaped Particle Damping Tuned Mass Damper (C-type PD-TMD). Using a dual-particle model and particle swarm optimization for parameter design, they verified the PD-TMD’s superior performance over conventional TMDs under large displacements and various excitations, demonstrating broader damping bandwidth and enhanced robustness.

In summary, conventional tuned mass dampers (TMD) have become inadequate for modern offshore wind structure vibration control due to their limited robustness and narrow bandwidth. The harsh marine environment and complex dynamic loads impose stricter requirements on structural fatigue life, necessitating the development of novel damping devices. McNamara et al. [21] proposed multiple tuned mass dampers (MTMD), which employ multiple substructures to control different frequencies, thereby addressing TMD’ limitations in control stability and bandwidth. Xiao et al. [22,23] developed energy dissipation models for particle dampers (PD) through simulations applied to gear transmission systems and electric multiple-unit power packages, with experimental validation confirming their effectiveness and demonstrating PD’ significant vibration suppression potential. Lu et al. [24] conducted comparative studies on TMD and tuned particle dampers (PTMD) using a five-story steel frame model, finding that both significantly reduced structural displacement and acceleration responses when frequency-tuned, with PTMD exhibiting superior performance, broader frequency bandwidth, and enhanced robustness. Subsequently, they performed free and forced vibration tests (including seismic, wind, and coupled wind-seismic excitations) on wind turbine structures, showing that PTMD effectively reduced dynamic responses at the tower top and internodes, outperforming TMD [25]. Zhang et al. [26] experimentally investigated the control effectiveness of particle impact dampers on first-mode vibrations of a cantilevered steel cylinder. Results indicated significant multi-directional vibration suppression with a notably broader effective bandwidth than conventional TMD.

The Pendulum Tuned Mass Damper (PTMD) features a simple structure, primarily consisting of a mass unit, pendulum cords, elastic components, and damping elements. Under specific parameter designs, it can operate without auxiliary elastic parts. The system operates solely based on structural vibration to drive the mass block, requiring no additional sensors, control systems, or external power supply, which enhances its reliability, reduces maintenance costs, and provides certain multi-directional vibration control capabilities [27]. In harsh marine environments, the Particle Damper (PD)—a type of mass-type passive damping device—serves as the damping element in the PTMD. It exhibits high durability, reliability, insensitivity to temperature variations when metallic particles are used, simple construction, low cost, and minimal impact on the installed structure. To ensure corrosion resistance, marine-grade anti-corrosion coatings are applied to the damper surface, while stainless steel or ceramic particles are selected to enhance internal corrosion resistance. Salt spray testing of appropriate grades during manufacturing guarantees sufficient corrosion protection.

**Fig 1:**
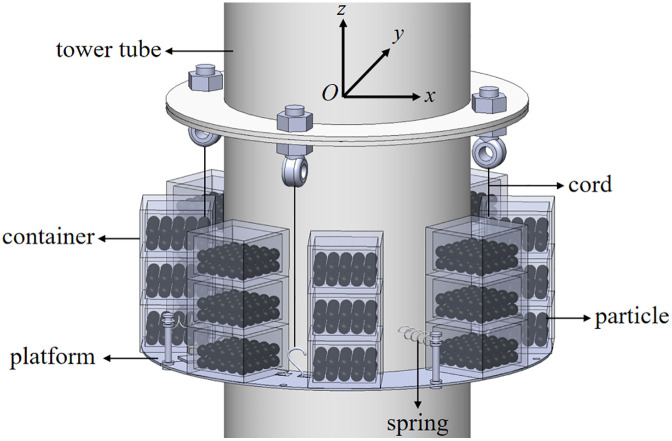
Schematic diagram of a PTPD.

**Fig 2:**
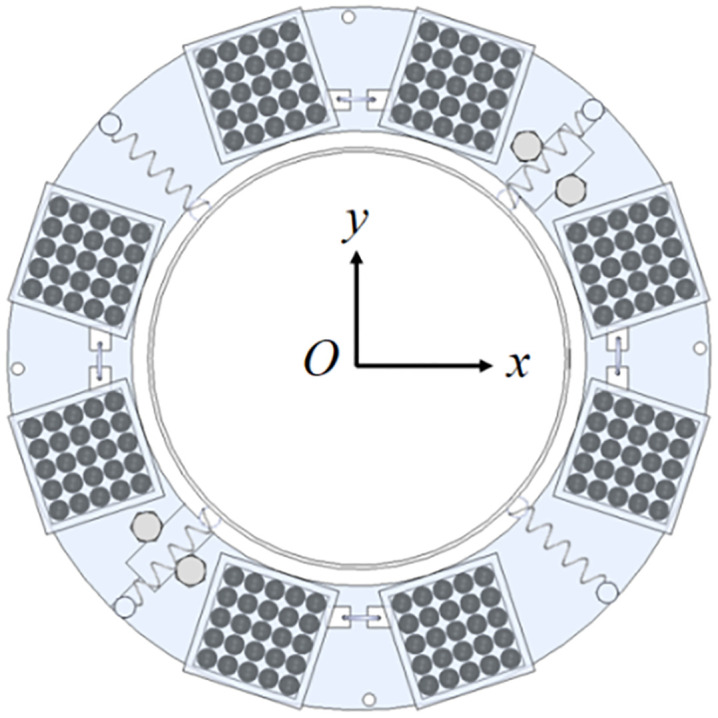
Top view of a PTPD.

Based on these advantages, this study integrates the PTMD and PD into a novel Pendulum-Tuned Particle Damper (PTPD), proposed for offshore wind turbine towers (Figs 1 and 2). The device consists of a support platform horizontally suspended by four equal-length cords and connected to the tower wall via tension springs, with customized particle-filled containers (adjustable in material, size, and mass) mounted on the platform. The vibration suppression mechanism of the PTPD is achieved through the rational design of the spring, chord length, and mass parameters of the damper, enabling frequency tuning to transfer the tower vibration energy induced by external excitation to the mass unit via inertial forces. This results in a counter-acting inertial force that reduces the dynamic response of the tower. The energy is subsequently dissipated through friction and collisions among the particles and between the particles and the container walls. To prevent alterations in the dynamic response caused by repeated disassembly of the scaled tower and to facilitate parameter adjustment during experimental validation, the PTPD was mounted on the external flanges. For full-scale tower structures, the PTPD can be installed on internal flanges to utilize the available interior space.

## 2. Mathematical modeling of PTPD for vibration suppression

### 2.1. Development of a simplified model for offshore wind turbine-damper systems

Offshore wind turbines are constantly subjected to external loads, yet their limited structural damping impedes vibration attenuation. Pendulum-tuned particle dampers installed at critical locations effectively suppress these vibrations.

**Fig 3:**
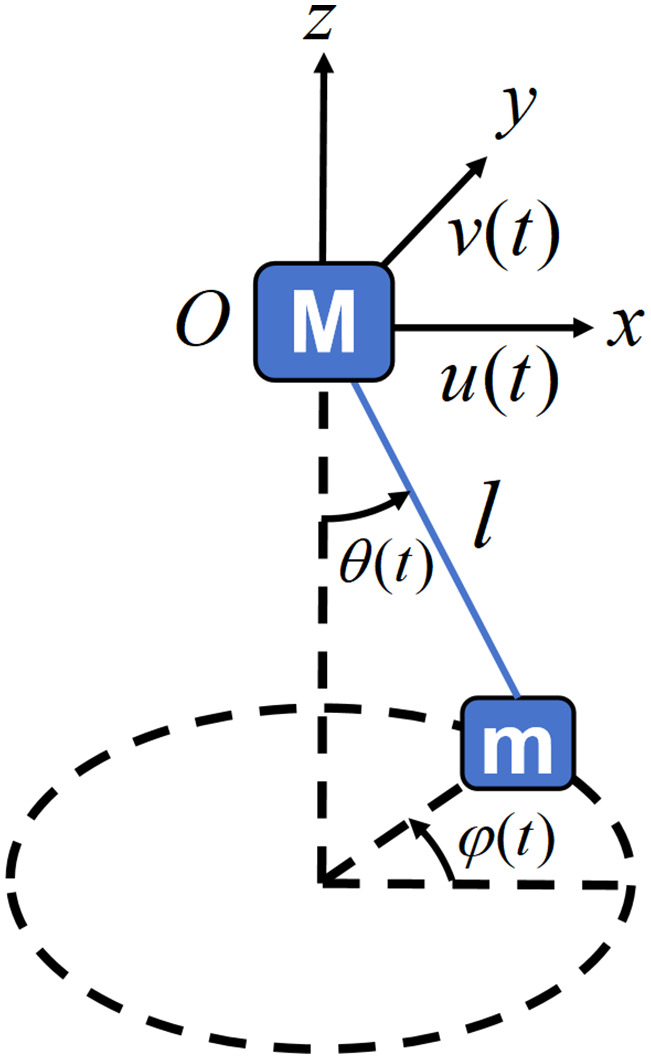
Schematic diagram of the main system-damper four-degree-of-freedom system.

This research focuses on vibration control for monopile-supported offshore turbines. The discrete nature of particle systems complicates modeling, necessitating initial analysis of a simplified particle-free model. Following the simplified modeling strategy in Reference [20], the offshore wind turbine is represented as a two-degree-of-freedom primary system, denoted as M in Fig 3. This simplification is adopted because the study focuses primarily on the vibration suppression performance of the PTPD itself rather than on detailed structural modeling of the wind turbine. Any errors introduced by this simplification are negligible for evaluating the PTPD’s damping performance, as they do not significantly affect the core analysis. Moreover, the simplified model remains capable of capturing key vibrational characteristics of the wind turbine system. The primary system exhibits two-degree-of-freedom motion in the xOy -plane, with displacements u(t) (denoted u_,_ x -direction) and v(t) (denoted v_,_ v -direction).

In previous studies on mathematical modeling and vibration control of PTMDs, the device is often idealized as a rigid pendulum rod, with one end hinged to the host structure and the other end fixed to a pendulum mass concentrated at its center of gravity [28–31]. For PTMD operating in a two-dimensional plane, the system can be simplified as a single-degree-of-freedom model, whereas a three-dimensional motion requires a two-degree-of-freedom representation [32]. Furthermore, Xu [33] demonstrated that when the rotation angle of the pendulum is less than approximately 9°, nonlinear effects can be neglected, allowing the system to be treated as linear. In this study, under the assumptions of negligible particle motion and linear system behavior, the four pendulum cords in Fig 1 are simplified as a single rigid pendulum rod. The platform, container, and particles are idealized as a concentrated pendulum mass m located at their collective center of mass, rigidly connected to one end of the pendulum rod. A horizontal linear spring and a linear viscous damper are incorporated between mass m and the tower at the same height. The other end of the pendulum rod is hinged at the center of the tower cross-section where the original four pendulum cords are attached. This yields a simplified 2-DOF system characterized by two generalized coordinates when particle motion is neglected: planar rotation angle θ(t) (denoted θ_,_ cord-to-vertical angle through origin O) and spherical rotation angle φ(t) (denoted φ_,_ box rotation about vertical axis).

**Fig 4:**
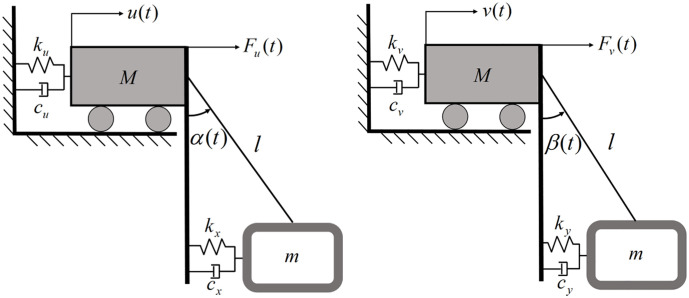
Plane mechanical modeling of the main system-damper system xOz vs. yOz.

Fig 4 presents the mechanical model of the primary-damper system in the xOz and yOz planes, showing forces $F_{u}(t)$ (x -direction) and $F_{v}(t)$ (y -direction) acting on the primary system. Parameter α(t) denotes the angle between the pendulum cord and the vertical line passing through point M projected onto the xOz plane at time t, while parameter β(t) represents the corresponding angle projected onto the yOz plane.

### 2.2. Lagrange-based equations of motion for the pendulum damper system

The Lagrange equation for the forced vibration of the primary-damper system is:


$$\frac{d}{dt}\frac{\partial T}{\partial {\dot{q}}_{r}}-\frac{\partial T}{\partial q_{r}}+\frac{\partial V}{\partial q_{r}}+\frac{\partial F}{\partial {\dot{q}}_{r}}=\mathrm{\backslash bold}Q_{r}$$ (1)

Where $q_{r}$ and ${\dot{q}}_{r}$ represent the generalized coordinates (u, v, θ, φ) and generalized velocities ($\dot{u}$, $\dot{v}$, $\dot{\theta }$, $\dot{\varphi }$), ${𝐐}_{𝐫}$ denotes the generalized force ${𝐐}_{𝐫}=\left[\;\;\;\begin{array}{cccc} {}*20cF_{u}(t) & F_{v}(t) & 0 & 0 \end{array}\;\;\right]$, and T, V, F correspond to the system’s kinetic energy function, potential energy function, and dissipation function, respectively.

#### 2.2.1. Kinetic energy function.

The position of the damper s(t) (denoted s) is defined by five parameters: planar angle θ, spherical angle φ, pendulum length l, primary system displacement u and v.


$$s=\left\{\;\;\;\begin{array}{c} {}*20cu+lsin\theta cos\varphi \\ v+lsin\theta sin\varphi \\ -lcos\theta \end{array}\;\;\;\right\}$$ (2)

The three rows in the vector above represent the damper’s position in x, y, z, differentiating with respect to time yields the damper’s velocity v(t) (denoted v):


$$v=\left\{\;\;\;\begin{array}{c} {}*20c\dot{u}+l\dot{\theta }cos\theta cos\varphi -l\dot{\varphi }sin\theta sin\varphi \\ \dot{v}+l\dot{\theta }cos\theta sin\varphi +l\dot{\varphi }sin\theta cos\varphi \\ l\dot{\theta }sin\theta \end{array}\;\;\;\right\}$$ (3)

Thus, the damper’s kinetic energy function is:


$$\begin{array}{cc} T_{m} & =\;\frac{\text{1}}{\text{2}}v^{T}mv\\ & =\;\frac{\text{1}}{\text{2}}m[(\dot{u}+\dot{\theta }lcos\theta cos\varphi -\dot{\varphi }lsin\theta sin\varphi {)}^{2}\\ & +\;(\dot{v}+\dot{\theta }lcos\theta sin\varphi +\dot{\varphi }lsin\theta cos\varphi {)}^{2}+(\dot{\theta }lsin\theta {)}^{2}]\\ & =\;\frac{\text{1}}{\text{2}}m({\dot{u}}^{2}+{\dot{v}}^{2}+l^{2}{\dot{\theta }}^{2}+l^{2}{\dot{\varphi }}^{2}sin^{2}\theta +2\dot{u}\dot{\theta }lcos\theta cos\varphi \\ & -\;2\dot{u}\dot{\varphi }lsin\theta sin\varphi +2\dot{v}\dot{\theta }lcos\theta sin\varphi +2\dot{v}\dot{\varphi }lsin\theta cos\varphi ) \end{array}$$ (4)

The primary system’s kinetic energy function is:


$$T_{M}=\frac{\text{1}}{\text{2}}{𝐕}^{T}\left[M\right]𝐕=\frac{\text{1}}{\text{2}}{\left\{\;\;\;\begin{array}{c} {}*20c\dot{u}\\ \dot{v} \end{array}\;\;\;\right\}}^{T}\left[\;\;\;\begin{array}{cc} {}*20cM_{uu} & M_{uv}\\ M_{vu} & M_{vv} \end{array}\;\;\;\right]\left\{\;\;\;\begin{array}{c} {}*20c\dot{u}\\ \dot{v} \end{array}\;\;\;\right\}$$ (5)

Where [M] is the mass matrix and 𝐕 is the velocity vector.

#### 2.2.2. Potential energy function.

The damper’s potential energy comprises gravitational potential energy $V_{g}$ and elastic potential energy $V_{k}$:


$$V_{g}=-mglcos\theta$$ (6)

Where g is gravitational acceleration.

The displacement increments $r_{x}$, $r_{y}$, $r_{z}$ at both ends of a linear spring in the x, y, z directions are:


$$r_{x}=lsin\theta cos\varphi$$ (7)

$$r_{y}=lsin\theta \sin\varphi$$ (8)

$$r_{z}=l(1-cos\theta )$$ (9)

With stiffness coefficients $k_{x}$, $k_{y}$, $k_{z}$ in the x, y, z directions, the elastic potential energy is:


$$\begin{array}{cc} V_{k} & =\frac{\text{1}}{\text{2}}k_{x}r_{x}^{2}+\frac{\text{1}}{\text{2}}k_{y}r_{y}^{2}+\frac{\text{1}}{\text{2}}k_{z}r_{z}^{2}\\ & =\frac{\text{1}}{\text{2}}l^{2}(k_{x}sin^{2}\theta cos^{2}\varphi +k_{y}sin^{2}\theta sin^{2}\varphi +k_{z}+k_{z}cos^{2}\theta -2k_{z}cos\theta ) \end{array}$$ (10)

The primary system’s potential energy function is:


$$V_{M}=\frac{\text{1}}{\text{2}}{𝐒}^{T}\left[K\right]𝐒=\frac{\text{1}}{\text{2}}{\left(\;\;\;\begin{array}{c} u\\ v \end{array}\;\;\;\right)}^{T}\left[\;\;\;\begin{array}{cc} {}*20ck_{uu} & k_{uv}\\ k_{vu} & k_{vv} \end{array}\;\;\;\right]\left\{\;\;\;\begin{array}{c} {}*20cu\\ v \end{array}\;\;\;\right\}$$ (11)

Where [K] is the stiffness matrix and 𝐒 is the displacement vector.

#### 2.2.3. Dissipation functions.

Energy dissipation in the damper system originates from damping elements. For linear viscous dampers with damping coefficients $c_{x}$, $c_{y}$, $c_{z}$ in the x, y, z directions:


$$\begin{array}{cc} F_{c} & =\frac{\text{1}}{\text{2}}c_{x}{\dot{r}}_{x}^{2}+\frac{\text{1}}{\text{2}}c_{y}{\dot{r}}_{y}^{2}+\frac{\text{1}}{\text{2}}c_{z}{\dot{r}}_{z}^{2}\\ & =\frac{\text{1}}{\text{2}}l^{2}[c_{x}(\theta^{2}cos^{2}\theta cos^{2}\varphi +{\dot{\varphi }}^{2}sin^{2}\theta sin^{2}\varphi -2\dot{\theta }\dot{\varphi }sin\theta sin\varphi cos\theta cos\varphi )\\ & +c_{y}({\dot{\theta }}^{2}cos^{2}\theta sin^{2}\varphi +{\dot{\varphi }}^{2}sin^{2}\theta cos^{2}\varphi +2\dot{\theta }\dot{\varphi }sin\theta sin\varphi cos\theta cos\varphi )+c_{z}{\dot{\theta }}^{2}\sin^{2}\theta ] \end{array}$$ (12)

The primary system’s dissipation function is:


$$F_{M}=\frac{\text{1}}{\text{2}}{𝐕}^{T}\left[C\right]𝐕=\frac{\text{1}}{\text{2}}{\left\{\;\;\;\begin{array}{c} {}*20c\dot{u}\\ \dot{v} \end{array}\;\;\;\right\}}^{T}\left[\;\;\;\begin{array}{cc} {}*20cC_{uu} & C_{uv}\\ C_{vu} & C_{vv} \end{array}\;\;\;\right]\left\{\;\;\;\begin{array}{c} {}*20c\dot{u}\\ \dot{v} \end{array}\;\;\;\right\}$$ (13)

Where [C] is the damping matrix.

#### 2.2.4. Governing equations for the primary-damper system.

The system’s kinetic energy function is:


$$T=T_{m}+T_{M}$$ (14)

The potential energy function is:


$$V=V_{g}+V_{k}+V_{M}$$ (15)

The dissipation function is:


$$F=F_{c}+F_{M}$$ (16)

Substituting Eqs. (14), (15), and (16) into Eq. (1) yields the system of governing equations for the 4-DOF primary-damper system:


$$\begin{array}{c} \left[\;\;\;\begin{array}{c} {}*20c\left[M\right]+\left[\;\;\;\begin{array}{cc} {}*20cm & 0\\ 0 & m \end{array}\;\;\;\right] \end{array}\;\;\;\right]\left(\;\;\;\begin{array}{c} \ddot{u}\\ \ddot{v} \end{array}\;\;\;\right)+\left[C\right]\left\{\;\;\;\begin{array}{c} {}*20c\dot{u}\\ \dot{v} \end{array}\;\;\;\right\}+\left[K\right]\left\{\;\;\;\begin{array}{c} {}*20cu\\ v \end{array}\;\;\;\right\}=\left\{\;\;\;\begin{array}{c} {}*20cF_{u}\\ F_{v} \end{array}\;\;\;\right\}\mathrm{\backslash vspace}2mm\\ +\;ml\times \left\{\;\;\;\begin{array}{c} {}*20c-\ddot{\theta }cos\theta cos\varphi +{\dot{\theta }}^{2}sin\theta cos\varphi +2\dot{\theta }\dot{\varphi }cos\theta sin\varphi +\ddot{\varphi }sin\theta sin\varphi +{\dot{\varphi }}^{2}sin\theta cos\varphi \\ -\ddot{\theta }cos\theta sin\varphi +{\dot{\theta }}^{2}sin\theta sin\varphi -\dot{\theta }\dot{\varphi }2cos\theta cos\varphi -\ddot{\varphi }sin\theta cos\varphi +{\dot{\varphi }}^{2}sin\theta sin\varphi \end{array}\;\;\;\right\} \end{array}$$ (17)

### 2.3. FEM-DEM coupled simulation

**Fig 5:**
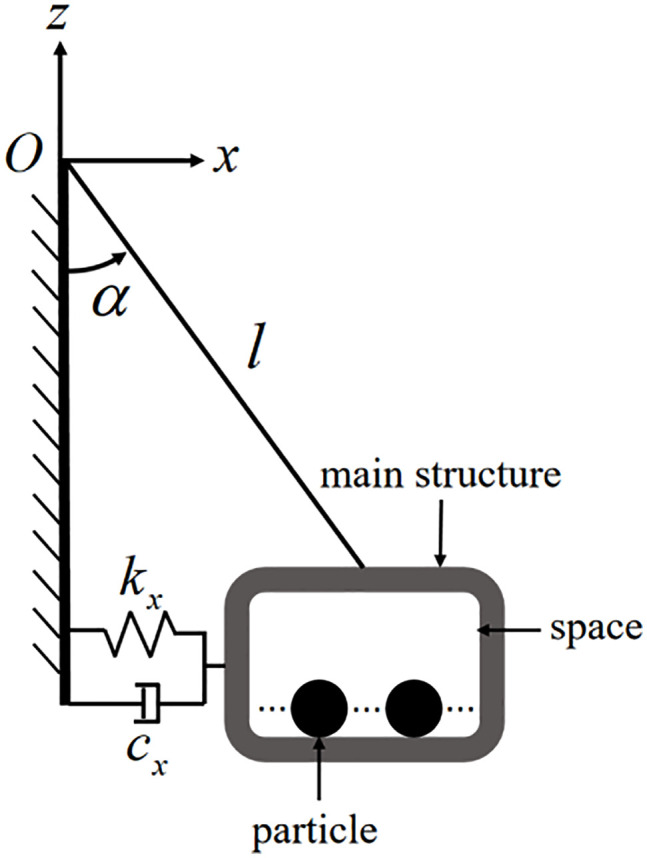
xOz planar mechanical model of the PTPD.

The primary-damper system’s operation involves particle-induced energy dissipation. However, the particle system’s discontinuity prevents Lagrangian formulation integration, precluding direct FEM computation of the damper’s damping matrix. Thus, a coupled FEM-DEM approach analyzes particle effects. Fig 5 shows the pendulum-tuned particle damper’s mechanical model in the xOz -plane (analogous to the yOz -plane model), with angle α representing the pendulum cord’s xOz -plane projection versus the vertical axis.

**Fig 6:**
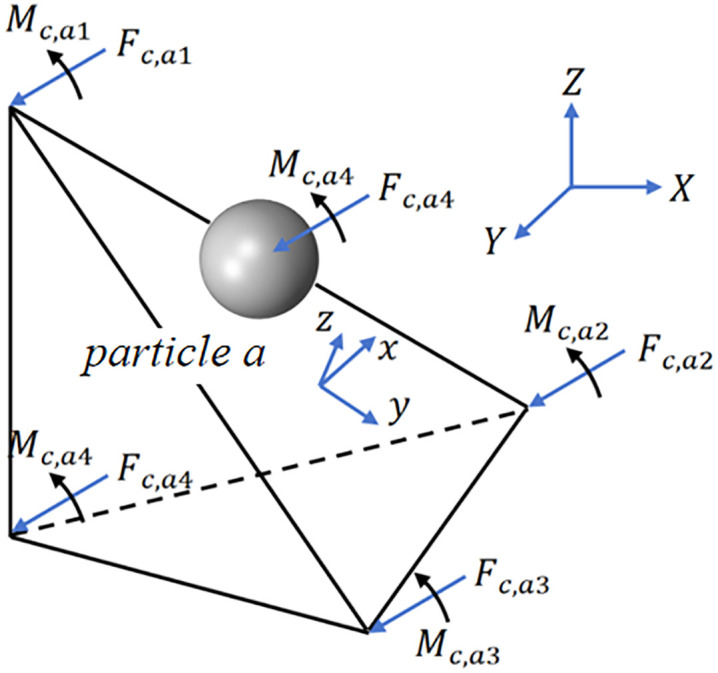
Triangle unit contact force/torque conversion diagram.

The pendulum damper’s container walls are discretized into triangular shell elements during simulation. Using the shape function method, loads computed by the DEM are transferred from individual elements to FEM nodes to enable coupled FEM-DEM calculation [34]. As shown in Fig 6, o−xyz represents the local coordinate system of a triangular element, a identifies a particle, $F_{c,a}$ and $M_{c,a}$ denote the force and moment vectors of particle a acting on the element plane and nodes respectively. $n_{x}=n_{ij},n_{y}=n\times n_{x},n_{z}=n$ indicates the axis vector of the element’s local coordinate system (where n is the unit normal vector, with $\bar{n}=n_{ij}\times n_{ik}/|n_{ij}\times n_{ik}|$, $n_{ik}$ and $n_{ij}$ being unit vectors connecting particle i to j and particle i to k respectively).

The displacement and rotation at any point within the element can be expressed in terms of nodal displacements and rotations:


$$\{n_{x},n_{y},n_{z},\theta_{x},\theta_{y},\theta_{z}{\}}^{T}=[N{]}_{6\times 18}\{n_{xi},n_{yi},n_{zi},\theta_{xi},\theta_{yi},\theta_{zi},n_{xj},\cdots ,\theta_{zj},n_{xk},\cdots ,\theta_{zk}{\}}^{T}$$ (18)

Where [N] relates nodal displacements/rotations to intra-element values: $n_{xi}$ represents node i ‘s x -direction displacement, $\theta_{xi}$ its x -direction rotation, $n_{x}$ the x -direction displacement at any intra-element point, and $\theta_{x}$ the corresponding rotation, with analogous definitions for other variables.

The relationship between the element’s local coordinate system and the global coordinate system is:


$$\{x,y,z{\}}^{T}=\left[\;\;\begin{array}{c} {}*20c{𝐓}_{t,1} \end{array}\;\;\right]\{X,Y,Z{\}}^{T}$$ (19)

Where $\left[\;\;\;\begin{array}{c} {}*20c{𝐓}_{t,1} \end{array}\;\;\;\right]$ is the transformation matrix, $\left[\;\;\;\begin{array}{c} {}*20c{𝐓}_{t,1} \end{array}\;\;\;\right]={\left[\;\;\;\begin{array}{c} {}*20c\left\{n_{x}\}\{n_{y}\}\{n_{z}\right\} \end{array}\;\;\;\right]}^{T}$.

The following transformation matrix converts the contact force and moment vectors from the local coordinate system to the global coordinate system:


$${\left[\;\;\;\begin{array}{c} {}*20c{𝐓}_{t,2} \end{array}\;\;\;\right]}_{6\times 6}=\left[\;\;\;\begin{array}{cc} {}*20c\left[\;\;\;\begin{array}{c} {}*20c{𝐓}_{t,1} \end{array}\;\;\;\right] & 0\\ 0 & \left[\;\;\;\begin{array}{c} {}*20c{𝐓}_{t,1} \end{array}\;\;\;\right] \end{array}\;\;\;\right]$$ (20)

Similarly, the following transformation matrix converts the forces and moments at the three nodes of the triangular element from the local coordinate system to the global coordinate system:


$${\left[\;\;\;\begin{array}{c} {}*20c{𝐓}_{t,3} \end{array}\;\;\;\right]}_{18\times 18}=\left[\;\;\;\begin{array}{ccc} {}*20c\left[\;\;\;\begin{array}{c} {}*20c{𝐓}_{t,2} \end{array}\;\;\;\right] & 0 & 0\\ 0 & \left[\;\;\;\begin{array}{c} {}*20c{𝐓}_{t,2} \end{array}\;\;\;\right] & 0\\ 0 & 0 & \left[\;\;\;\begin{array}{c} {}*20c{𝐓}_{t,2} \end{array}\;\;\;\right] \end{array}\;\;\;\right]$$ (21)

The contact forces on the nodes in the global coordinate system are:


$${\left\{{𝐅}_{c,particle}\right\}}_{18\times 1}=\sum_{a=1}^{M}{\left[{𝐓}_{t,3}\right]}_{18\times 18}^{T}{\left\{{𝐍}_{a}\right\}}_{18\times 6}^{T}{\left[{𝐓}_{t,2}\right]}_{6\times 6}^{T}{\left\{{𝐖}_{c,a}\right\}}_{6\times 1}$$ (22)

Where ${𝐍}_{a}$ is the interpolation matrix of the element contacted by particle a, M is the number of particle contact points acting on the triangular element, and ${𝐖}_{c,a}$ represents the force and moment vectors exerted by particles on the triangular element.

Based on the above formulations, the particle forces and moments acting on the container shell elements are transformed into equivalent nodal forces at corresponding positions, thereby enabling the determination of the damping force exerted by particles on the pendulum motion.

## 3. Computational analysis of vibration suppression using a PTPD

### 3.1. Numerical investigation of damper parameter effects on vibration control performance

The natural frequency $\omega_{a}$ of the pendulum damper is:


$$\omega_{a}=\sqrt{\frac{mgl+kh^{2}}{ml^{2}}}$$ (23)

Where h denotes the length from the connection point of the auxiliary stiffness device to the suspension point. When h = l, the following relation holds:


$$\omega_{a}=\sqrt{\frac{g}{l}+\frac{k}{m}}$$ (24)

For an undamped primary system with pendulum damper coupling, the closed-form solution minimizing the primary system’s RMS response yields optimal tuning parameters: frequency ratio $f_{opt}$ and damping ratio $\zeta_{opt}$ [35]. This solution assumes: concentrated bob mass at the pendulum connection, negligible cord mass, and cord length substantially exceeding bob dimensions. The expressions are:


$$f_{opt}=\frac{\omega_{aopt}}{\omega_{n}}=\frac{\sqrt{1+\frac{\mu }{\text{2}}}}{1+\mu }$$ (25)

$$\zeta_{opt}=\sqrt{\frac{\mu +\frac{3\mu^{2}}{\text{4}}}{4+6\mu +2\mu^{2}}}$$ (26)

Where $\omega_{n}$ is the undamped natural frequency of the primary system, and μ is the mass ratio defined as $\mu =\frac{m}{M}$.

**Fig 7:**
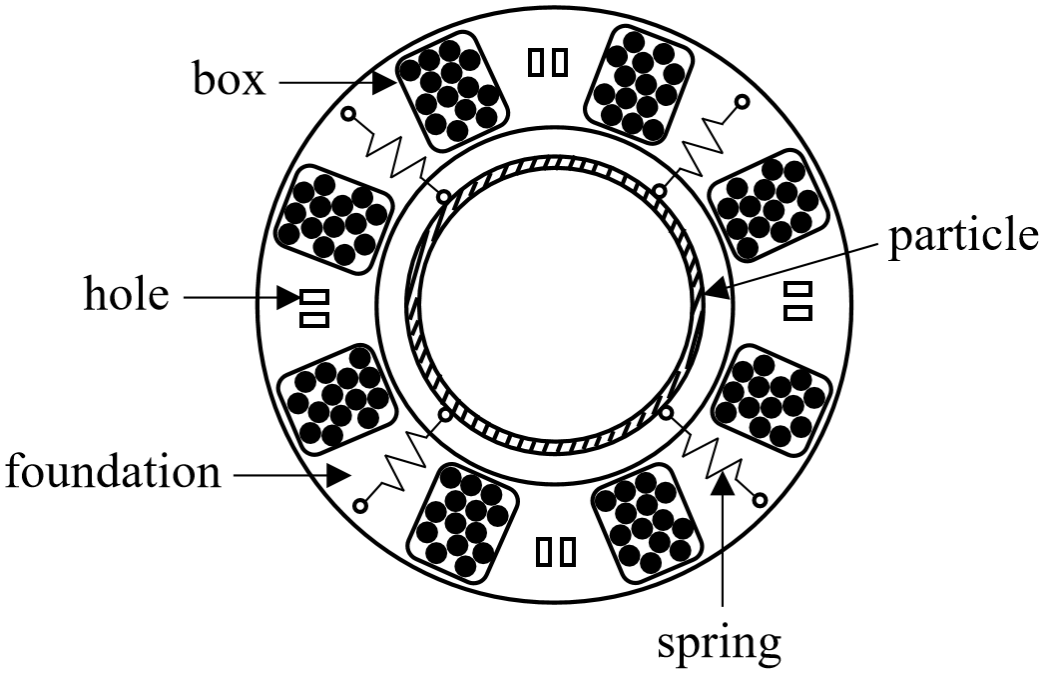
Relative position of damper components.


When designing a dampers for offshore wind turbines with known natural frequency and mass, a suitable mass ratio μ (typically 0.01–0.1) is first selected. The corresponding optimal frequency ratio $f_{opt}$ is derived from Equation (25), obtaining the damper’s optimal natural frequency $\omega_{a}$. An appropriate chord length l is then chosen considering installation spatial constraints. Substituting into Equation (24) yields the damper stiffness coefficient k. In the implemented pendulum-tuned particle damper, four springs are symmetrically arranged on a ring (Fig 7). With minimal length variations relative to initial spring length $l_{0}$ during vibration, the composite stiffness becomes [26]:

$$k_{z}=\left(4-\frac{2l_{0}}{l_{0}+\Delta l}\right)k$$ (27)

Where Δl is the initial elongation.

The vibration control system targets axial vibrations at the tower top. A simplified SDOF turbine model (Fig 4) reduces computational demands while eliminating interference. To accommodate laboratory spatial constraints, a scaled wind turbine model was designed to a 1:65 scale using dimensional similarity analysis. Length, stress, and acceleration were selected as the controlling scaling factors, with values set to 1/65, 1, and 2, respectively. Although wind-induced tower vibrations typically range from 0.3 to 0.6 Hz (1st/2nd natural frequencies) [36], scaling limitations necessitate adjusting the SDOF system’s natural frequency to 0.85 Hz for experimental validation. Key system parameters of the simplified SDOF turbine model include: mass M = 25 kg, stiffness k = 713.08 N/m, and damping coefficient c = 6.52 N·s/m.

**Fig 8:**
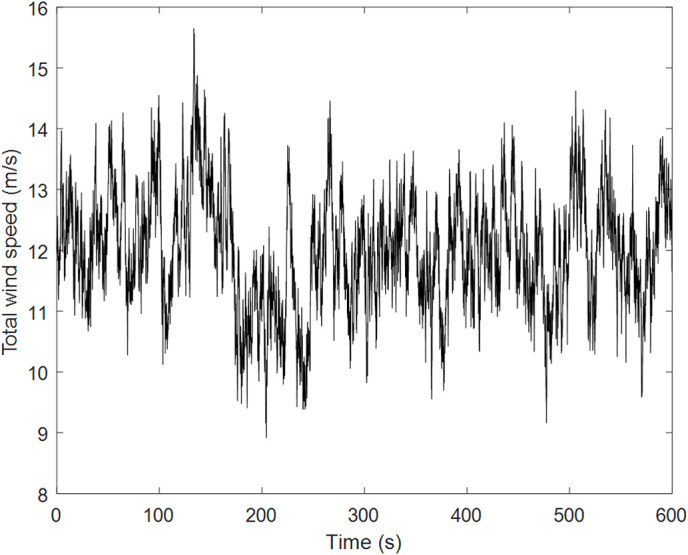
Pulsating wind speed map with average wind speed of 12m/s over 600s of time course.


Sea-surface wind speed comprises mean and turbulent components. Following prototype conditions, the Kaimal spectrum is adopted. Fig 8 displays the turbulent wind time history at 12 m/s mean speed, with corresponding power spectral density:

$$S_{vv}(h,f)=\frac{u_{*}^{2}}{f}\frac{200c}{{\left(1+50c\right)}^{5/3}}$$ (28)

Where c is the Monin coordinate, f is frequency, h is the height for wind load calculation, and $u_{*}$ is the friction velocity.

**Fig 9:**
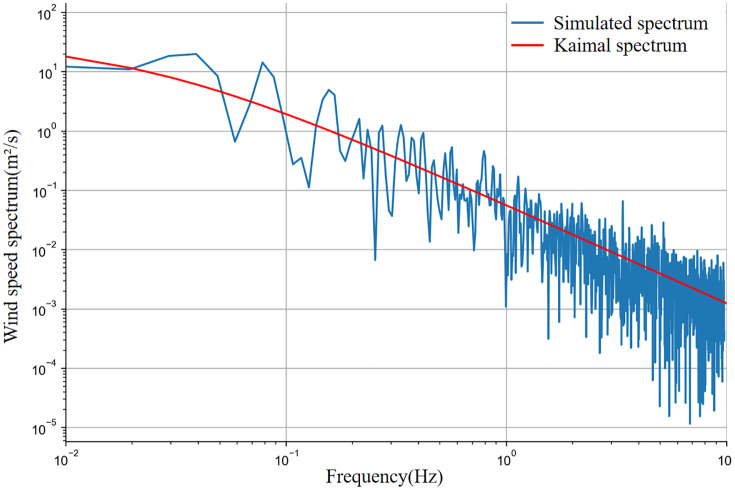
Wind speed spectrum comparison.

Fig 9 compares the simulated and target wind speed spectra, demonstrating the close agreement of the simulated wind load.

Wind loads on the turbine comprise tower pressure $F_{1}$, rotor axial load $F_{t}$, rotor torque $M_{r}$, and pitching moment $M_{n}$, expressed as:


$$F_{1}=\frac{\text{1}}{\text{2}}\rho A_{t}{V_{h}}^{2}C_{e}$$ (29)

$$F_{t}=\frac{\text{1}}{\text{2}}\rho A_{b}V^{2}C_{t}$$ (30)

$$M_{r}=\frac{9950P\gamma }{𝛺}$$ (31)

$$M_{n}=\frac{4\rho }{27B}\pi R^{3}\left(V_{2}^{2}-V_{1}^{2}\right)$$ (32)

Where $V_{h}$ is the wind speed at height h, ρ is air density, $A_{t}$ is the tower windward area, $C_{e}$ is the drag coefficient, $A_{b}$ is the swept area, $C_{t}$ is the thrust coefficient, P is generator power, γ is efficiency, Ω is rotor speed, B is the blade count, and $V_{1}$ / $V_{2}$ are wind speeds at the upper/lower 2/3 blade length from the hub center.

**Fig 10:**
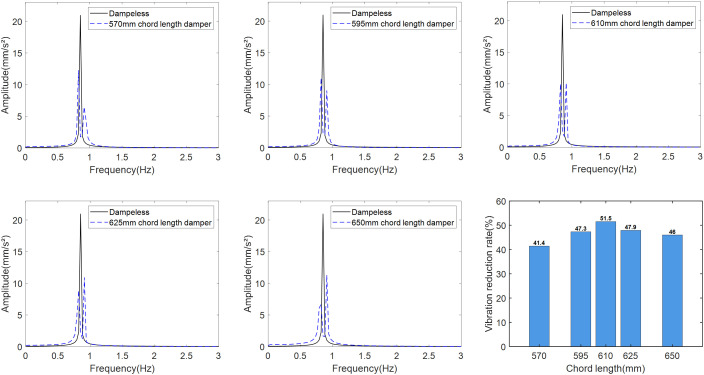
Damping spectrum and damping rate for each chord length damper. (a) Chord length 570 mm. (b) Chord length 595 mm. (c) Chord length 610 mm. (d) Chord length 625 mm. (e) Chord length 650 mm. (f) Damping rate of each chord length damper.

Eq. (24) demonstrates that suppressing the original tower vibration peak under turbulent wind requires only chord length optimization when bob mass and spring stiffness remain constant. Simulation analysis with a 315 g bob and 5 N/m spring stiffness evaluates five chord lengths: 570, 595, 610, 625, and 650 mm (Fig 10 acceleration spectra).

The tower vibration acceleration spectra for varying chord lengths reveal asymmetric dual peaks, with the left peak exceeding the right peak when the chord length is shorter than 610 mm. This indicates partial but suboptimal vibration reduction because the damper’s tuned frequency deviates from the primary tower vibration peak at 0.85 Hz. A comparable detuning effect appears when the chord length surpasses 610 mm. The optimal parameter combination consists of: 315 g bob mass, 5 N/m spring stiffness, and 610 mm chord length.

### 3.2. Parametric simulation and optimization of PTPD for vibration control

This section investigates how particle material and size affect PTPD performance. The model consolidates eight particle boxes into a single unit, with S4R shell elements for the container and homogeneous PD3D units (identical, 6-DOF) for particles, following Hertzian contact theory.

**Fig 11:**
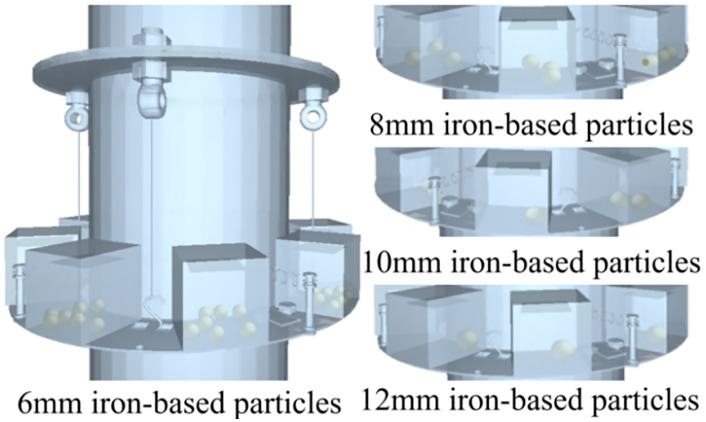
Iron-based particle damper filling chart.

**Fig 12:**
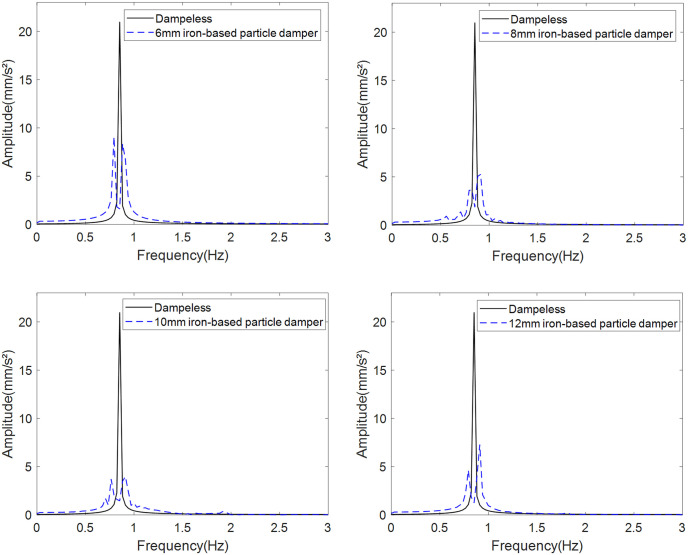
Vibration damping effect of dampers filled with different particle sizes of iron-based particles.

To isolate material and size effects, the container mass is fixed at 265 g with 50 g total particle mass. Simulations evaluate peak acceleration reduction in the frequency domain for iron-based particles (6–12 mm diameter) compared to baseline vibration (Figs 11 and 12).

Comparison of peak accelerations from the four spectra with the baseline vibration (Table 1) reveals that 10 mm iron-based particles provide optimal vibration reduction (81.1%). Increasing particle diameter reduces quantity but enhances energy dissipation per particle-wall collision. The 10 mm diameter achieves optimal balance between dissipation per event and collision frequency, maximizing vibration reduction.

**Table 1:** The influence of iron-based particle size on the suppression rate.

Particle size	6mm	8mm	10mm	12mm
Suppression rate	51.2%	74.9%	81.1%	65.2%

**Fig 13:**
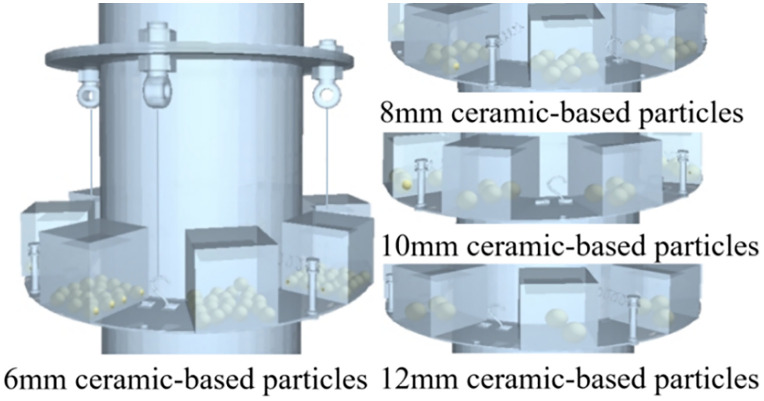
Ceramic-based particle damper filling diagram.

**Fig 14:**
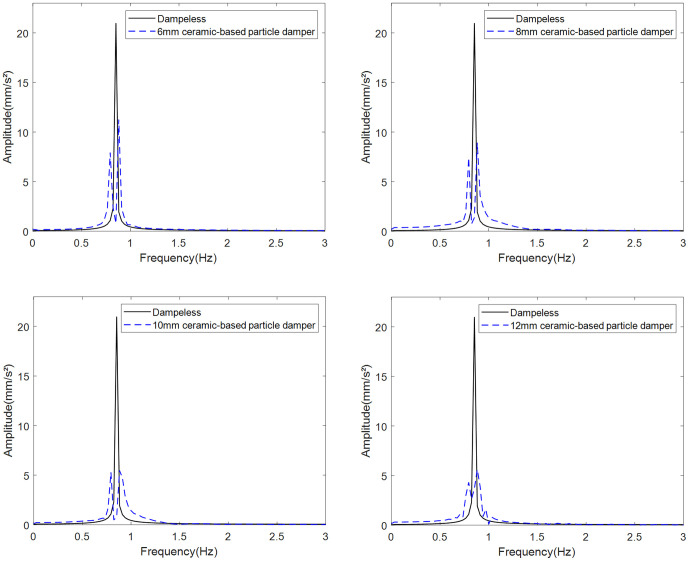
Vibration damping effect of dampers filled with ceramic-based particles of different particle sizes.

Using identical mass conditions (265 g container, 50 g particles), simulations assess vibration reduction for ceramic-based particles with diameters of 6–12 mm (Figs 13 and 14).

Comparative analysis with the baseline vibration peak (Table 2) demonstrates that 10 mm ceramic-based particles achieve 73.9% vibration attenuation. However, their reduced density relative to iron-based particles decreases collision energy dissipation, resulting in 12.2% lower efficiency than iron-based counterparts. The higher density of iron-based particles further permits 18–22% more compact damper configurations at equivalent mass loading. Consequently, 10 mm iron-based particles demonstrate superior overall performance.

**Table 2:** The influence of ceramic-based particle size on the inhibition rate.

Particle size	6mm	8mm	10mm	12mm
Suppression rate	46.3%	57.4%	67.5%	73.2%

## 4. Experimental analysis of multi-parameter vibration suppression performance in a PTPD

### 4.1. Baseline vibration analysis of wind turbine towers

#### 4.1.1. Scaled prototype of wind turbine system.

The 8 MW offshore wind turbine prototype incorporates a monopile-supported tower constructed from Q345 structural steel. The four-segment tower measures 95.96 m total height (segment lengths: 13.8 m, 25.2 m, 25.2 m, 31.76 m), transitioning from cylindrical to conical geometry with diameters decreasing from 6.5 m at the base to 5.02 m at the top, and wall thicknesses reducing from 60 mm to 25 mm. The complete tower structure weighs 495 tonnes. The nacelle-hub assembly, measuring 19.5 m in length, 8.3 m in width, and 13.9 m in height, has a mass of 452.5 tonnes, while each 85.6 m blade weighs 32.5 tonnes.

#### 4.1.2. Design methodology for scaled wind turbine models.

A 1:65 scale model with 1 mm uniform wall thickness was designed for FEM-DEM validation against experimental data, accounting for fabrication constraints (Table 3). Flange-welded segment ends allow bolted assembly. The nacelle contains a bearing-supported shaft that extends forward to connect with the three-blade hub.

**Table 3:** Model tower parameters(units:mm).

	First paragraph	Second paragraph	Third paragraph	Fourth paragraph
Bottom diameter	100	100	100	100
Top diameter	100	100	100	80
Lengths	210	380	380	480

#### 4.1.3. Sensor network configuration design.

**Fig 15:**
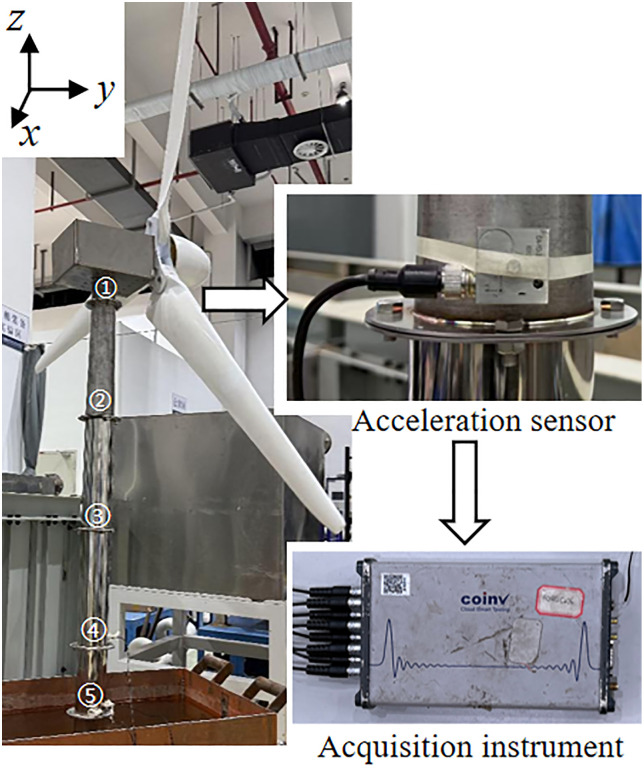
Layout of measurement points and sensors.

Triaxial accelerometers are magnetically mounted at five tower locations (P1-P5) above flange connections (Fig 15) to monitor horizontal vibration profiles across different elevations. The model of the signal collector is INV3062T0, and the model of the acceleration sensor is CA-YD-3193T-A.

#### 4.1.4. Baseline vibration spectrum analysis of scaled tower models.

**Fig 16:**
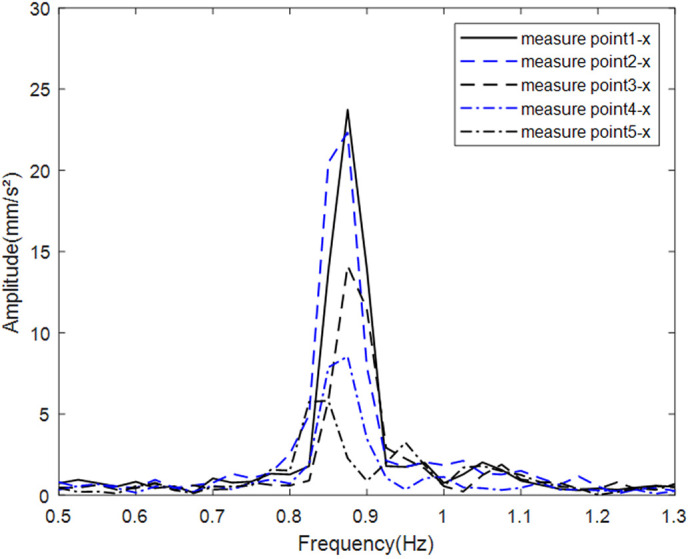
Raw vibration spectra of tower tube x-axis at each height measurement point.

**Fig 17:**
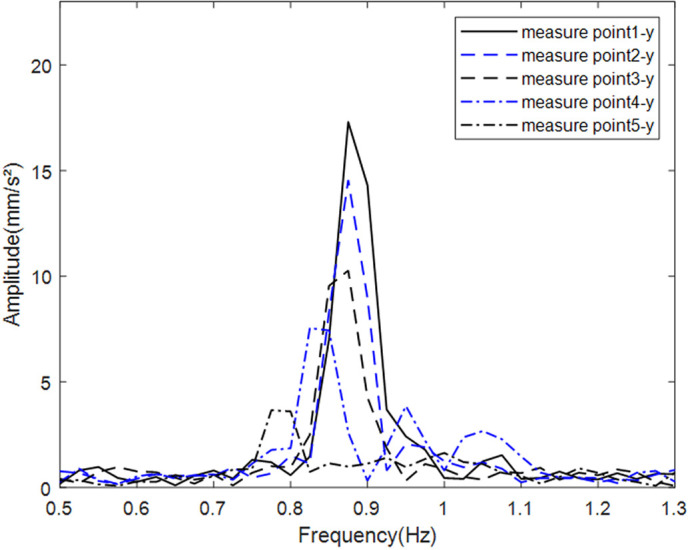
Raw vibration spectra of tower tube y-axis at each height measurement point.

Due to laboratory equipment constraints, simulated wind loads were generated by an airflow generator installed in front of the scaled wind turbine model to drive the blades. After stable operation was achieved, vibration signals at measurement points P1–P5 under simulated wind loads were recorded at different rotational speeds. The results indicate that when the blade speed reached 50 r/min, the blade-passing frequency approached the fundamental frequency of the tower. Peak acceleration responses in both the x and y directions occurred at the first-order natural frequency of 0.85 Hz (Figs 16 and 17). Furthermore, the amplitude decreased with increasing tower height, and the x -direction vibration consistently exceeded the y -direction response at the same elevation. These observations suggest that the dynamic response in the upper part of the tower is primarily influenced by the blade-passing frequency and wind load. Owing to the exterior mounting of the PTPD on the tower and the use of acrylic material for the particle containers, the motion behavior of both the PTPD and the internal particles could be observed in real time during the experiments.

### 4.2. Effect of damper configuration on vibration suppression performance

For large-scale structures, multiple small tuned mass dampers offer installation, maintenance, and cost advantages over single large units. In wind turbines, multiple dampers increase the effective mass ratio, improving vibration suppression. Tower vibration responses were analyzed for one (P1 flange), two (P1-P2 flanges), and three (P1-P3 flanges) damper configurations at optimal mass ratio, using P1 acceleration data to evaluate axial vibration changes.

**Fig 18:**
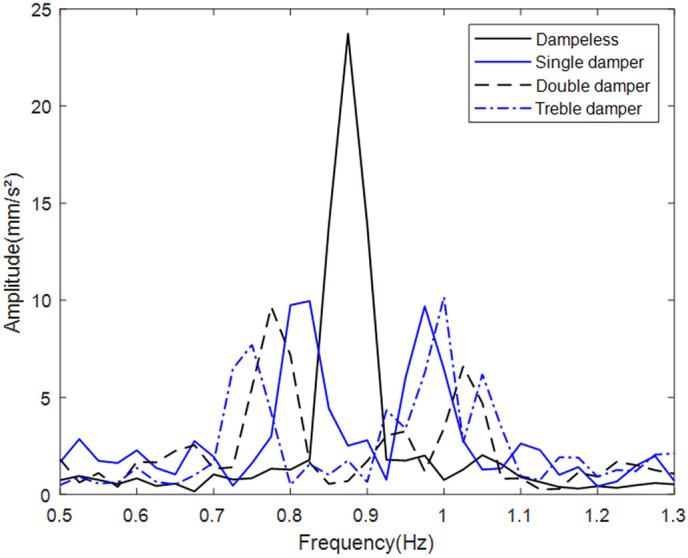
Comparison of the vibration damping effects of single, double and triple dampers.

Fig 18 spectral analysis demonstrates significant upper-tower amplitude attenuation using a single damper. A second damper yields marginal additional reduction but slightly widens the suppression bandwidth, likely due to the scaled model’s low baseline vibration limiting dual-damper gains. A third damper provides minimal amplitude improvement while further extending the effective frequency band. Table 4 details peak suppression rates for all configurations.

**Table 4:** The suppression rates of different numbers of dampers.

Arrangement	Single damper	Double damper	Triple damper
Suppression rate	58.0%	59.2%	57.1%

### 4.3. Influence of particle filling characteristics on damper effectiveness

**Fig 19:**
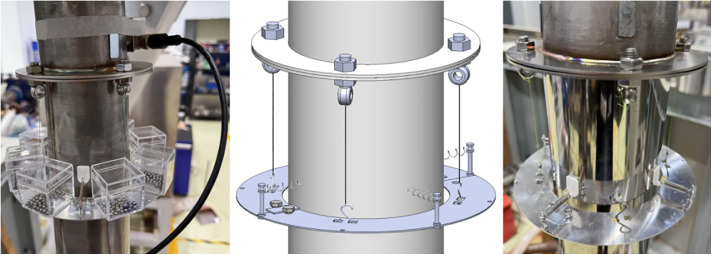
Schematic diagram of a pendulum damper.

Eight particle boxes were symmetrically arranged on the damper support platform (Fig 19) to compare vibration reduction performance across filling schemes. Each box contained identical particles in material, diameter and mass, with total mass equally distributed according to the optimal ratio. Using a three-damper configuration with P1 monitoring, the variation in damping performance was analyzed when the containers were filled with 8 mm, 10 mm, and 12 mm iron-based particles, ceramic-based particles, and equivalent solid masses.

The suppression rates of acceleration curve peaks for different particles are detailed in Table 5.

**Table 5:** The suppression rates of dampers filled with different materials.

Filler	Particle size	Ceramic-based particles	Iron-based particles	lron blocks of the same mass
Suppression rate	8mm	62.6%	64.7%	59.2%
	10mm	67.9%	72.0%	
	12mm	68.0%	67.8%	

**Fig 20:**
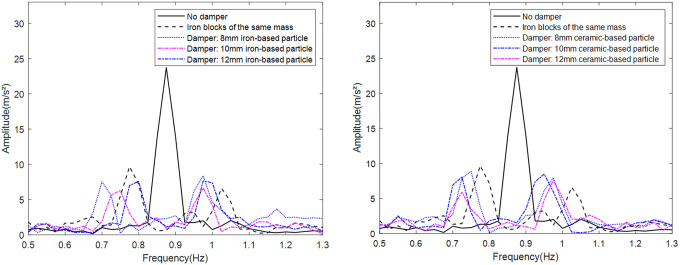
Comparison of vibration damping effect of different particle dampers.

Fig 20 presents the vibration acceleration spectra of the tower equipped with dampers filled with iron-based and ceramic-based particles of various sizes. The vibration suppression performance of all particle-filled dampers exceeded that of dampers using equivalent solid masses under identical mass conditions. A detailed analysis of dampers filled with the same material but different particle sizes revealed that the highest vibration reduction rates—68.0% and 72.0%—were achieved with 12 mm ceramic particles and 10 mm iron particles, respectively. As particle size increased, the performance of ceramic-based particle dampers improved and eventually stabilized, whereas iron-based dampers exhibited a non-monotonic trend (initially decreasing, then increasing, and later decreasing again), indicating the presence of an optimal configuration under the given conditions. Comparison among dampers filled with equal-size particles of different materials showed that iron-based particles outperformed ceramic-based ones, likely due to their higher individual mass, which enhances energy dissipation per collision—particularly advantageous under low-frequency vibration where impact events are less frequent. The experimental results align well with simulation outcomes, validating the numerical approach adopted in this study.

**Fig 21:**
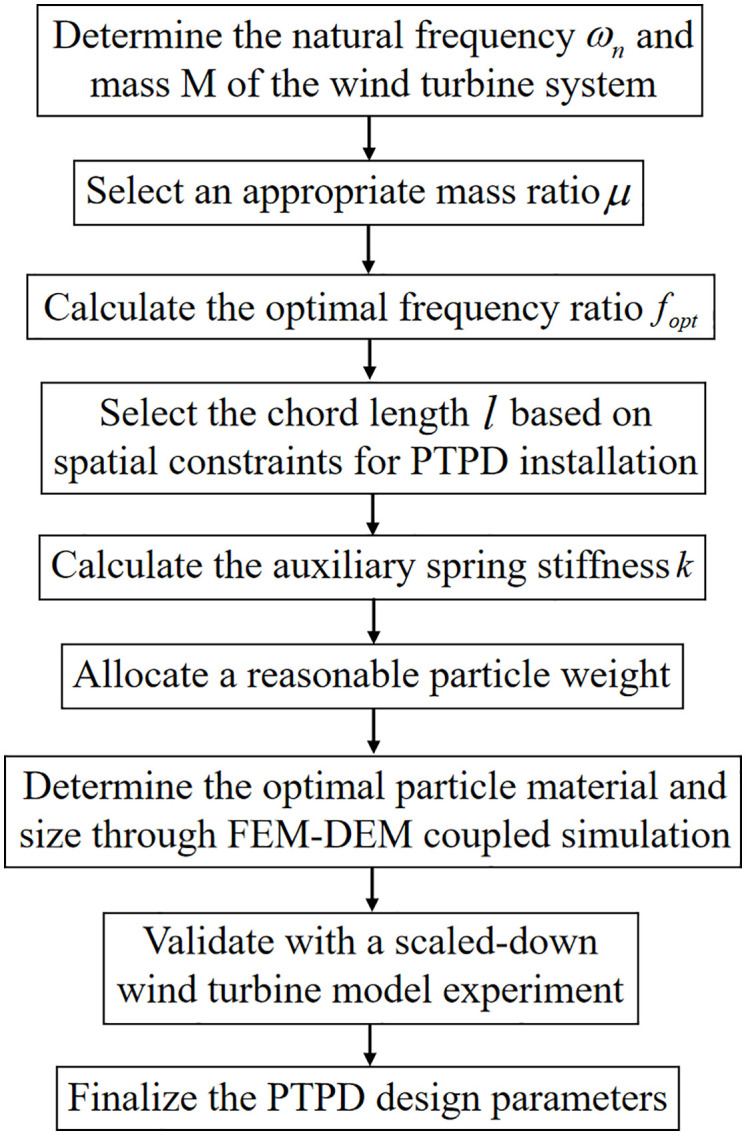
The schematic design of the PTPD.

Fig 21 illustrates the schematic design of the PTPD for suppressing wind-induced vibrations in offshore wind turbine towers.

## 5. Conclusion

This study proposes and systematically evaluates a novel Pendulum Tuned Particle Damper (PTPD) for suppressing vibrations in offshore wind turbine towers. Through integrated theoretical modeling, finite element–discrete element (FEM-DEM) coupled simulations, and experimental validation on a scaled tower model, the vibration reduction mechanism and performance optimization of the PTPD were thoroughly investigated.

(1) A simplified four-degree-of-freedom mathematical model of the wind turbine–PTPD system was established using Lagrange’s equations. Simulations analyzing the influence of chord length variation on the damper’s vibration suppression performance identified the optimal chord length for a PTPD with a mass of 315 g and stiffness of 5 N/m.
(2) The nonlinear energy dissipation behavior induced by particle collisions and friction was simulated using coupled FEM-DEM simulations. Experimental results demonstrated good agreement with numerical outcomes, confirming the accuracy of the simulation method. The study indicates that particle material and size significantly affect damping performance. Iron-based particles with a diameter of 10 mm were identified as the optimal filling material, achieving a vibration reduction rate of 81.1%, superior to ceramic-based particles of the same size. Experiments also confirmed that higher particle density enhances energy dissipation per collision, making iron-based particles more suitable for controlling low-frequency vibrations with infrequent impact events.
(3) The vibration suppression effect of a distributed damper configuration was examined. While a single damper effectively reduced vibration amplitude, a distributed layout provided considerable vibration suppression while substantially reducing the size and weight of individual dampers, offering a more compact and practical design solution for real-world engineering applications.
